# Stromal Expression of MiR-21 Predicts Biochemical Failure in Prostate Cancer Patients with Gleason Score 6

**DOI:** 10.1371/journal.pone.0113039

**Published:** 2014-11-17

**Authors:** Christian Melbø-Jørgensen, Nora Ness, Sigve Andersen, Andrej Valkov, Tom Dønnem, Samer Al-Saad, Yury Kiselev, Thomas Berg, Yngve Nordby, Roy M. Bremnes, Lill-Tove Busund, Elin Richardsen

**Affiliations:** 1 Department of Medical Biology, UIT The Arctic University of Norway, Tromsø, Norway; 2 Department of Clinical Medicine, UIT The Arctic University of Norway, Tromsø, Norway; 3 Department Oncology, University Hospital of North Norway, Tromsø, Norway; 4 Department of Clinical Pathology, University Hospital of North Norway, Tromso, Norway; 5 Department of Pharmacy, UIT The Arctic University of Norway, Tromsø, Norway; 6 Department of Urology, University Hospital of North Norway, Tromsø, Norway; Yokohama City University School of Medicine, Japan

## Abstract

**Aim:**

microRNAs (miRNAs) are involved in various neoplastic diseases, including prostate cancer (PCs). The aim of this study was to investigate the miRNA profile in PC tissue, to assess their association with clinicopathologic data, and to evaluate the potential of miRNAs as diagnostic and prognostic markers.

**Materials and Methods:**

From a cohort of 535 patients submitted to radical prostatectomy (RP), a sample of 30 patients (14 patients with rapid biochemical failure (BF) and 16 patients without BF) with Gleason score 7 were analyzed. A total of 1435 miRNAs were quantified by microarray hybridization, and selected miRNAs with the highest Standard deviation (n = 50) were validated by real-time quantitative PCR (qRT-PCR). *In situ* hybridization (ISH) was used to evaluate the expression of miR-21.

**Results:**

miR-21 was the only miR that was significantly up-regulated in the BF group (p = 0.045) miR-21 was up-regulated in patients with BF compared with non-BF group (p = 0.05). In univariate analyses, high stromal expression of miR-21 had predictive impact on biochemical failure-free survival (BFFS) and clinical failure-free survival (CFFS) (p = 0.006 and p = 0.04, respectively). In the multivariate analysis, high stromal expression of miR-21 expression was found to be an independent prognostic factor for BFFS in patients with Gleason score 6 (HR 2.41, CI 95% 1.06–5.49, p = 0.037).

**Conclusion:**

High stromal expression of miR-21 was associated with poor biochemical recurrence-free survival after RP. For patients with Gleason score 6, miR-21 may help predict the risk of future disease progression and thereby help select patients for potential adjuvant treatment or a more stringent follow-up.

## Introduction

Prostate cancer (PC) is the second leading cause of cancer-related death among males [1]. The disease outcome is variable and difficult to predict. During the last 30 years, the number of radical prostatectomies (RP) has increased 25-fold, mainly due to patients overdiagnosed with nonlethal cancer [2]. Testing for prostate-specific antigen (PSA) is the most common tool to detect prostate cancer. However, recent studies have shown that PSA concentrations are unable to differentiate between indolent and life-threatening cancers at the time of diagnosis [3]. An identification of better prognostic markers for risk stratification will therefore have major impact on the clinical management of PC.

miRNAs constitute a class of small non-coding RNA molecules (∼20 nucleotides) that are involved in regulating protein expression. miRNAs can be produced as a by-product from mRNA production as inter-intron passengers, or can be transcribed as a single- or polycistronic product by RNA polymerase II [4]. They work by binding to the 3′ UTR of the target mRNA, and induce silencing of the mRNA by the Argonaut (Ago) protein in the RNA-induced Silencing protein complex (RISC) [4], [5]. Many miRNAs are deregulated in cancer and influence on tumor formation and progression because they are located in regions of the genome that are commonly overexpressed or deleted [6]. Several miRNAs and their targets are expressed abnormally in PC, leading to tumor progression, invasion, and metastasis. The altered expressions of some selected miRNAs are potentially useful as biomarkers for diagnosis, prognosis, and classification purposes of PC [7]–[9]. miR-21 was the first oncogenic miRNA to be discovered [10]. In PC, miR-21 is considered to act as an oncogene, but its role is unclear, and the reports are conflicting. Hulf et al. [11] found miR-21 to act as a tumor suppressor gene, while Ribas et al. [12] reported that overexpression of miR-21 promoted both hormone-dependent and hormone-independent tumor growth in PC cell lines. Moreover, they concluded that elevated levels of miR-21 increases tumor development, tumor growth and induced castration-resistant phenotype [13]. In contrast, Folini et al. [14] did not find any differences in miR-21 expression between normal prostate tissue and PC. Shi et al. [15] found miR-21 to be involved in chemoresistance and that miR-21 was up-regulated in Docetaxel resistant PC3 (PCR3) cells compared to wild type PC3 cells.

In this study, we investigated the miRNA profile in PC patients. Among 1435 miRNAs, miR-21 was the only candidate miRNA that was significantly up-regulated and underwent further evaluation as a prognostic marker for the entire cohort. The Regional Committee for Medical and Health Research Ethics (2009/1393), the Data Protection Official for Research (NSD), and the National Data Inspection Board have approved this study. The ethics committee waived the need for consent. The patient records was anonymized and de-identified prior to analysis.

## Patients and Methods

### Patients and tissue samples

Primary tumor tissue from 535 radical prostatectomy (RP) patients diagnosed at the University Hospital of Northern Norway, St. Olav Hospital and Nordland Hospital from 1995–2005 were used in this study. Adequate paraffin embedded tissue blocks and complete demographic and clinicopathological data were obtained for all patients (Table 1). The tumors were graded according to the modified Gleason grading system [16] and staged according to the WHO guidelines [17]. All primary tissues were histologically reviewed by two pathologists (ER and LTB).

**Table 1:** Patient characteristic, clinicopathological variables and their prognostic value for BF, CF, and PCD (univariate analyses; log rank test) (N = 535).

Characteristic	Patients (n)	Patients (%)	BF (170 events)		CF (36 events)		PCD (15 events)	
			5-year EFS (%)	P	10-year EFS (%)	P	10-year EFS (%)	P
**Age**				0.55		0.085		0.600
≤65 years	357	67	76		92		97	
>65 years	178	33	70		88		96	
**pT-stage**				**<0.001**		**<0.001**		**0.027**
pT2	374	70	83		96		98	
pT3a	114	21	60		86		98	
pT3b	47	9	43		73		89	
**Preop PSA**				**<0.001**		0.085		0.061
PSA<10	308	57	80		93		99	
PSA>10	221	42	67		88		95	
Missing	6	1	-		-		-	
**Gleason**				**<0.001**		**<0.001**		**0.001**
3+3	183	34	83		98		99	
3+4	220	41	76		93		98	
4+3	80	15	69		84		95	
4+4	19	4	63		76		94	
>8	33	6	34		67		87	
**Tumour Size**				**<0.001**		**0.019**		0.098
0–20 mm	250	47	82		94		99	
>20 mm	285	53	67		88		96	
**PNI**				**<0.001**		**<0.001**		**0.002**
N	401	75	79		95		98	
Yes	134	25	60		81		93	
**PSM**				**0.041**		**0.038**		0.697
N	249	47	81		94		97	
Yes	286	53	69		89		97	
**Circumferent PSM**				**<0.001**		**<0.001**		**0.029**
N	381	71	81		95		98	
Yes	154	29	57		81		94	
**Apical PSM**				**0.04**		0.484		0.31
N	325	61	73		90		96	
Yes	210	39	77		92		98	
**Vasc inf**				**<0.001**		**<0.001**		**0.009**
N	492	92	77		93		98	
Yes	43	8	46		71		88	
**Surgical proc**				0.23		0.41		0.581
Retropubic	435	81	76		90		97	
Perineal	100	19	67		95		98	

Abbreviations: BF = biochemical failure; CF = Clinical failure; EFS = event free survival in months; PCD = prostate cancer death; NR = not reached; P = P value for log rank statistic for difference in event free survival; PC = Prostate cancer; PNI = Perineural infiltration; Post op RT = postoperative radiotherapy; Preop = preoperative; PSA = Prostate specific antigen; PSM = Positive surgical margin; Surgical proc = surgical procedure; Vasc inf = Vascular infiltration.

### miRNA screening

For miRNA profiling, 30 patients within the Gleason Score (GS) 7 subgroup were consecutively chosen, 14 of them with a rapid biochemical failure BF (PSA≥0.4 ng/mL within 24 months post-surgery) and 16 patients with no BF. GS 7 was chosen as these patients show a heterogenic clinical outcome. The miRNAs were identified by microarray hybridization and quantified with RT-qPCR (Table 2). A total of 1435 miRNAs were quantified by microarray hybridization and selected miRNAs with the highest standard deviation (n = 50) were validated by quantitative real-time PCR (qRT-PCR). Seven miRNAs were identified as the most up- or down-regulated. Of these, miR-21 was the only candidate miR that was significantly up-regulated by fold-change. MiR-21 expression was then investigated in the entire cohort by chromogen *in* *situ* hybridization (cISH) and evaluated as a prognostic marker for PC. Both tumor epithelial cells and tumor associated stromal areas were investigated separately. The median follow-up time was 89 months (range 6–188). The last patient update was November 2012.

**Table 2:** Clinical details of patient samples used in the miRNA expression array (N = 30).

Sample ID	Age	Gleasonscore	t-size (mm)	pT-stage	Time to BF	CF	PNI	Vasc. Inf.
BF_1	65	3+4	10	pT2a	5.8	N	N	N
BF_2	62	3+4	30	pT2c	2	N	Yes	Yes
BF_3	62	4+3	34	pT3b	8.3	N	Yes	Yes
BF_4	60	4+3	40	pT3a	12.8	N	N	N
BF_5	69	4+3	22	pT2b	13.3	Yes	N	N
BF_6	65	3+4	23	pT2c	13.3	N	N	N
BF_7	64	3+4	19	pT2c	13.7	N	N	N
BF_8	70	3+4	26	pT3a	2	N	Yes	Yes
BF_9	69	3+4	19	pT2c	15.8	N	N	N
BF_10	68	4+3	13	pT2a	17	N	N	N
BF_11	58	3+4	40	pT3a	18.4	N	N	N
BF_12	59	4+3	17	pT2c	5.8	Yes	Yes	Yes
BF_13	66	3+4	43	pT3b	12.4	Yes	Yes	Yes
BF_14	70	3+4	30	pT3b	1.4	N	N	N
N_BF_1	56	3+4	20	pT3a	NA	NA	N	N
N_BF_2	61	3+4	10	pT2	NA	NA	N	N
N_BF_3	69	4+3	25	pT3a	NA	NA	Yes	N
N_BF_4	67	3+4	26	pT3a	NA	NA	N	N
N_BF_5	66	4+3	42	pT3a	NA	NA	N	N
N_BF_6	66	4+3	15	pT2c	NA	NA	N	N
N_BF_7	57	3+4	17	pT2c	NA	NA	N	N
N_BF_8	66	3+4	13	pT2c	NA	NA	N	N
N_BF_9	71	3+4	15	pT3b	NA	NA	N	N
N_BF_10	67	3+4	37	pT3a	NA	NA	Yes	N
N_BF_11	63	3+4	29	pT3a	NA	NA	N	N
N_BF_12	65	3+4	10	pT2a	NA	NA	N	N
N_BF_13	71	3+4	16	pT2b	NA	NA	N	N
N_BF_14	64	4+3	15	pT2a	NA	NA	N	N
N_BF_15	64	3+4	25	pT2b	NA	NA	Yes	N
N_BF_16	65	3+4	31	pT2a	NA	NA	Yes	N

Abbreviations: BF; biochemical failure, CF; clinical failure, PNI; perinural infiltration, Vasc. Infiltr: vascular infiltration.

### Microarray construction

Tissue Microarray (TMA) construction was chosen for high-throughput molecular pathology analysis [18]. For each case, a pathologist (ER) histologically identified and marked two cores with areas of tumor cells (epithelial tumor cells), two cores with tumor stromal tissue, one core from areas with normal epithelial cells, and one core with normal stromal tissue. The TMAs were assembled using a tissue-arraying instrument (Beecher Instruments, Silver Springs, MD, USA). Briefly, we used a 0.6 mm diameter needle to harvest the marked tissue areas from the corresponding formalin-fixed paraffin-embedded (FFPE) tissue blocks. The samples were inserted into an empty recipient paraffin block according to a coordinate pattern. To include all core samples, twelve tissue array blocks were constructed. Multiple 4 µm sections were cut with a Micron microtome (HM355S), affixed to glass slides, and sealed with paraffin. The detailed methodology has been reported previously [19].

### RNA extraction

RNA was isolated from formalin-fixed paraffin-embedded samples with RecoverAll Total Nucleic Acid Isolation Kit for FFPE tissue (Life Technologies), and was sent to Exiqon (Vedbaek, Denmark) which performed the miRNA screening experiment. Tissue used for RNA extraction was three 4 mm deep, 0.6 mm diameter cylindrical cores harvested from marked tumor compartments from FFPE blocks. The microarray used was LNA array 6^th^ generation human, rat and viral microarray designed with a miRNA library with 1435 human, rat and viral miRNA. Total RNA (500 ng) from both sample and reference was labeled with Hy3 and Hy5 fluorescent label, respectively, using the miRCURY LNA microRNA Hi-Power Labeling Kit, Hy3/Hy5 (Exiqon, Denmark) following the procedure described by the manufacturer. The Hy3-labeled samples and a Hy5-labeled reference RNA sample were mixed pair-wise and hybridized to the miRCURY LNA microRNA Array 6th Gen (Exiqon, Denmark), which contains capture probes targeting all microRNAs for human, mouse or rat registered in the miRBASE 16.0. The hybridization was performed according to the miRCURY LNA microRNA Array Instruction manual using a Tecan HS4800 hybridization station (Tecan, Austria). After hybridization, the microarray slides were scanned and stored in an ozone free environment (ozone level below 2.0 ppb) in order to prevent potential bleaching of the fluorescent dyes. The miRCURY LNA microRNA Array slides were scanned using the Agilent G2565BA Microarray Scanner System (Agilent Technologies, Inc., USA) and the image analysis was carried out using the ImaGene 9 (miRCURY LNA microRNA Array Analysis Software, Exiqon, Denmark). The quantified signals were background corrected and normalized using the global Lowess (LOcally WEighted Scatterplot Smoothing) regression algorithm.

### Quantification of mature miRNAs by real-time qPCR

The criteria for selecting miRNAs for PCR validation was; significant P-value, high fold change and prior plausibility. The miRNAs chosen for further testing were miR-21, miR-141, miR-23a, miR-222, miR-205, miR-143 and miR-145 (Table 2). All real-time qPCR experiments were performed by Exiqon (Vedbaek, Denmark).

RNA (2 µl) was reverse transcribed in 10 µl reactions using the miRCURY LNA Universal RT microRNA PCR, Polyadenylation and cDNA synthesis kit (Exiqon). cDNA was diluted 100 x and assayed in 10 µl PCR reactions according to the protocol for miRCURY LNA Universal RT microRNA PCR; each microRNA was assayed in triplicate by qPCR on the microRNA custom made pick-n-mix panel. Negative controls excluding template from the reverse transcription reaction was performed and profiled like the samples. The amplification was performed in a LightCycler 480 Real-Time PCR System (Roche) in 384 well plates. The amplification curves were analyzed using the Roche LC software, both for determination of Crossing point (Cp) by the 2nd derivative method, and for melting curve analysis.

The amplification efficiency was calculated using algorithms similar to the LinReg software. All assays were inspected for distinct melting curves and the melting temperature (T_m_) was checked to be within known specifications for the assay. Furthermore assays was detected by 5 Cp’s less than the negative control, and with Cp<37 to be included in the data analysis. Data that did not pass these criteria were omitted from any further analysis.

Using NormFinder the best normalizer was found to be the miR-23b. All data was normalized to this miRNA in all samples (miR-23b – assay Cp).

### *In situ* hybridization

Chromogen *in* *situ* hybridization (cISH) was performed according to “One-day microRNA ISH protocol” developed by Exiqon, Vedbek, Denmark. Labelled locked nucleic acid (LNA) modified probes from Exiqon for miR-21 (hsa-miR-21miRCURY LNA Prod. No. 38102-15), positive control (U6 has/mmu/rno) and negative controls (scramble-miRNA) was used. Some optimizations of the method were done to get a specific and sensitive detection of miRNA in our sections from FFPE TMAs.

4 µm TMA slides were incubated for three days at 37°C to attach cores to super Frost Plus slides. Sections were deparaffinised in xylene (3×5 min) and then hydrated to ethanol solutions to PBS, pH 7.4. Proteinase-K 20 µm/ml, treatment was done in PK-buffer (5 mM Tris-HCL, pH 7.5, 1 mM NaCl, autoclaved) at 37°C for 20 min in a ThermoBrite hybridizer. After PBS wash the sections were rehydrated through ethanol and air-dried. The LNA-probes were denatured by heating to 90°C for 4 min. Hybridization of the LNA-probes miR-21 (50 nM), scrambled miRNA (50 nM) and U6 (1 nM) were carried out in a ThermoBrite hybridizer at 50°C for 60 min. Stringent washes were performed in room temperature 5x SSC buffer, pre-heated SSC buffers (50°C), 5 min in 5x SSC, 2×5 min in 1x SSC, 2×5 min in 0.2 SSC, and 5 min in RT 0.2x SSC. Sections were blocked against unspecific binding in blocking solution from DIG wash and Block buffer set (11 585 762 001, Roche, Mannheim, Germany) for 15 min at RT in a humidity chamber. Alkaline phosphatase (AP)-conjugated anti DIG (11 093 274 910, Roche, Mannheim, Germany) 1∶800 was incubated for 30 min at RT in a humidity chamber for immunologic detection. After PBS-T wash the substrate enzymatic reactions was carried out with NBT/BCIP (11 697 471 001, Roche, Mannheim, Germany) at 30°C in the ThermoBrite for 120 min. The reaction was stopped with a 2×5 min wash in KTBT buffer (50 nM Tris-Hcl, 150 nM NaCl, 10NM KCI) followed by wash in double distilled water. Sections were counter stained with nuclear fast red (WALDECK, ZE-012-250) at RT for 1 min and then rinsed in tap water. Dehydration was accomplished by increasing gradients of ethanol solutions and finally mounting with Histokitt mounting medium (Assistant-Histokitt, 1025/250 Sondheim/Rhoen Germany).

### Scoring of cISH

The image analysis was performed using the ARIOL imaging system (Genetix, San Jose, USA) composing of a microscope (Olympus BX 61) equipped with an automatic stage and slide loader, together with a camera. The cores were photographed using 20x magnifications. The dominant staining intensity in epithelial tumor cells and tumor surrounding stromal cells were scored as; 0 = negative, 1 = weak, 2 = moderate and 3 = strong. All samples were anonymized and independently scored by two pathologists (ER and AV). When assessing one variable for a given core, the observers were blinded to scores of the other variables and to the outcome. Mean score for each case was calculated from all four cores and both examiners. In case of disagreement (score discrepancy >1), the slides were re-examined and consensus was reached by the observers.

### Statistical methods

All Statistical analyses were performed using the statistical package IBM SPSS, version 21 (SPSS Inc., Chicago, IL, USA) (S1). Scoring values from each pathologist were compared for inter-observer reliability by use of a two-way random effect model with absolute agreement definition. A Wilcoxon signed rank test was used to assess if it was a statistically significant difference in the expression of miR-21 between normal tissue and cancer tissue. When comparing the two sample groups (BF versus non-BF group) from the qPCR results, two-sided student’s t-test was used. For the entire cohort, Pearson chi-square tests and Spearman’s Correlation test were performed to examine associations between miR-21 expression and clinicopathological markers. The Kaplan-Meyer method was used to make plots of BFFS and CFFS. Log-rank test was used to test for statistical significance. Significant variables from the univariate analyses were further assessed in the multivariate survival analysis using a backward stepwise Cox regression model with a probability for stepwise entry removal at 0.05 and 0.10, respectively. The significance level used was p<0.05 for all analyses. All survival analyses were carried out using three different end-points: (i) biochemical failure free survival (BFFS), (ii) clinical failure free survival (CFFS), and (iii) PC death free survival (PCDFS). BF was characterized as a PSA≥0.4 ng/mL and rising in a minimum of two different blood samples postoperatively. CF was defined as verified local symptomatic progression and/or verified metastasis to bone, visceral organs or lymph nodes on CT, MR, bone scan or ultrasonography. PCDFC was defined as death caused by progressive PC.

## Results

### Patients’ characteristics and clinicopathological variables

#### Total cohort

An overview of the patient cohort’s demographic, clinical and histopathological characteristics is presented in Table 1. Median age at surgery was 62 (range 45–75). The prostatectomies were retropubic in 435 cases and perineal in 100 cases. At last follow-up, 170 patients had experienced BF, 36 CF, and 15 patients were dead of PC.

#### miRNA screening subgroup

In the 30 patients with Gleason score 7 who underwent miRNA screening, median age was 65 years (range 57–71), median PSA 18.8 ng/mL (range 5–79), median tumor size 22 mm (range 3–45), 21% experienced CF and 14% were dead of prostate cancer. In the total group of patients with Gleason score 7 (n = 270), median age was 62 (range 47–74), median PSA 18.0 ng/mL (range 0.7–71), median tumor size 23 mm, 22% experienced CF and 9% were dead of prostate cancer. None of these differences were statistically significant.

### Microarray screening and qPCR validation

**Figure 1:**
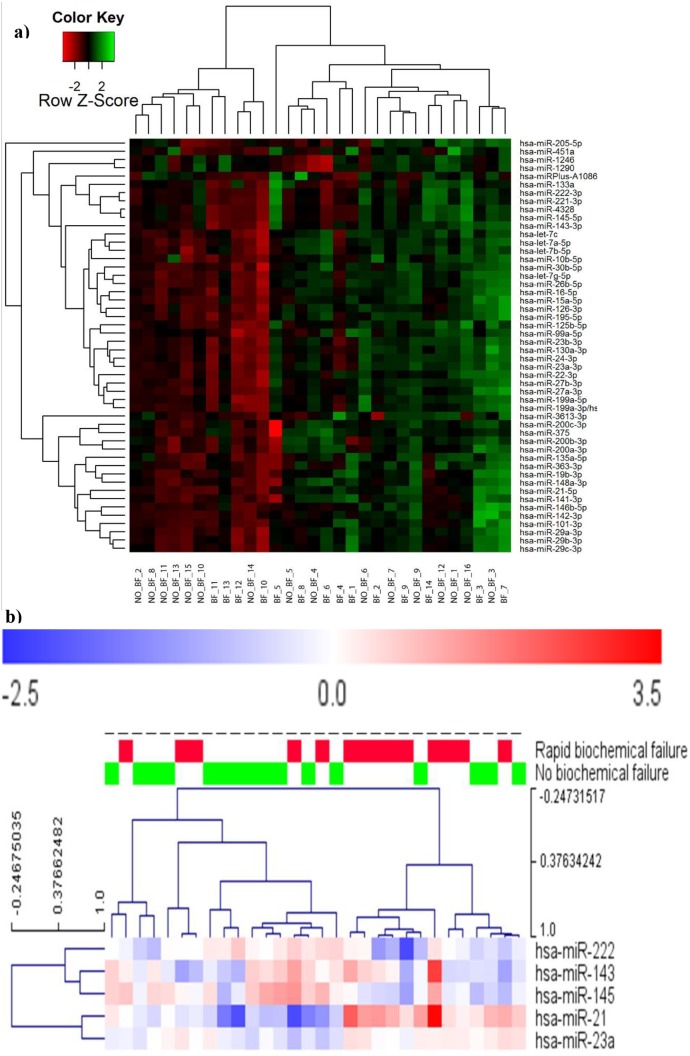
Linkage hierarchical unsupervised clustering of differentially expressed miRNAs. a) Relative expression levels of 50 miRNAs that were expressed in all 30 matched tissue samples with Gleason score 7. The X-axis shows individuals and the Y-axis shows miRNAs. Red squares encode for up-regulated miRNAs and green squares encode for down-regulated miRNAs. b) Heat Map results of the two-way hierarchical clustering based on the qPCR results. The miRNAs showed the same trend in qPCR validation and in the microarray analysis. The normalized (dCp) values have been used for the analysis. Red color represents an expression level above mean, blue color represents expression lower than the mean.

600 of 1435 miRNAs investigated by microarrays, had expression above background (background signal strength defined as 1.2 times the intensity of the 1-quartile each slide) (Table 2). Of these, 50 miRNAs with the highest standard deviation (SD) were further analyzed. From the microarray analyses, a hierarchical 2D-clustering showed a relative expression level of miRNAs that were up-regulated in both groups (Figure 1a). Expression levels of 7 miRNAs were validated by RT-qPCR (Table 3, Figure 1a). Five of seven analyzed miRNAs showed similar trend in the two groups. The Heat Map diagram shows the result of the two-way hierarchical clustering of the expression level of the five miRNAs. The z-score was cropped from −2 to +2 (Figure 1b). miR-21 was the only miRNA that was significantly up-regulated in the BF group. This was in concordance with the array results. There was a strong correlation between the array hybridization and qRT-PCR.

**Table 3:** The most up- or down-regulated miRNAs in rapid BF group versus non-BF group.

	Microarray			RT-qPCR	
	miR	Fold change	*p*	Fold change	*p*
Up-regulated	*hsa*-miR-141*	0.42	0.32	1.09	0.08
	*hsa*-miR-143	–0.42	0.34	0.10	0.74
	*hsa*-miR-21	0.31	0.46	0.89	0.05
	*hsa*-miR-23a	–0.28	0.38	0.12	0.34
Down-regulated	*hsa*-miR-145	–0.62	0.31	–0.15	0.58
	*hsa*-miR-205	–0.70	0.31	–0.48	0.68
	*hsa*-miR-222	–0.33	0.30	–0.22	0.35

Two-sided Student’s t-test: miR-21 was significant (cut-off: p-value<0.5).

*One-sided Student’s t-test: miR-141 was significant. Abbreviations: BF; biochemical failure.

### Expression of miR-21 and correlations in the total cohort

**Figure 2:**
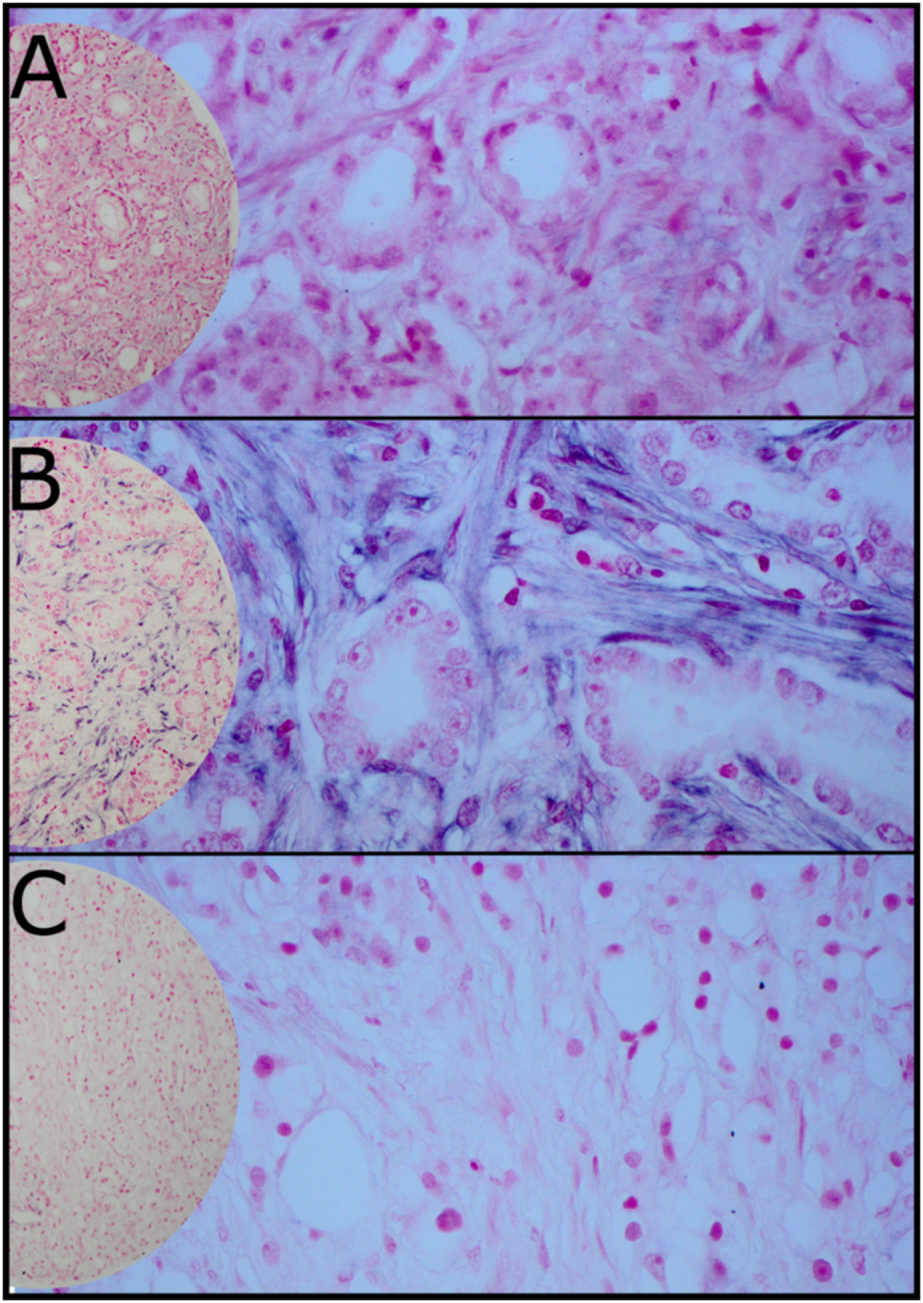
In situ hybridization (ISH) analysis of prostate cancer representing Gleason Grade (GS) <7 (A), GS 7 (B) and GS<7 (C), (200x and 400x magnification).

In general, miR-21 was expressed at a higher level in tumor stromal areas than in tumor epithelial cells (Figure 2). The intensity of miR-21 expression was stronger in cytoplasm of tumor tissue compared to normal tissues from the patients (p<0.001). There was no difference in miR-21 expression between tumor epithelial cells and normal epithelial cells. A significant higher expression of miR-21 was found in tumor stromal area than in non-neoplastic stromal area (p<0.001). By comparing tumor epithelial cells with tumor stromal cells, miR-21 was expressed at higher levels in tumor stromal cells (p<0.001).

There were significant correlations between high tumor stromal expression of miR-21 and pT-stage (*r* = 0.134, p = 0.003), perineural infiltration (PNI) (*r* = 0.175, p<0.001) and vascular infiltration (*r* = 0.222 p<0.001). We found a weak correlation between stromal expression of mir-21 and Gleason score (*r* = 0.218, p<0.001). There were also significant correlations between stromal expression of miR-21 and Gleason Score (GS); GS <7, GS 7 and GS>7 (r = 0.238, p<0.001).

There were no differences in stromal expression of miR-21 between the subgroups of Gleason score, 3+4 (n = 220, 41%) and 4+3 (n = 80, 15%) (r = – 0.001, p = 0.991).

### Univariate analysis

The clinicopathological variables pT-stage (p<0.001), preoperative PSA value dichotomized at 10 ng/dL (n = 221, p<0.001), Gleason score (p<0.001), tumor size dichotomized at 20 mm (n = 285, p<0.001), perineural infiltration (PNI) (no = 134, p<0.001), positive surgical margins (PSM) (n = 286, p = 0.041), positive surgical circumferential margin (n = 154, p<0.001), positive surgical apical margin (n = 210, p = 0.04), and vascular infiltration (n = 43, p<0.001) were all significantly correlated to BFFS in the univariate survival analyses (Table 1).

**Figure 3:**
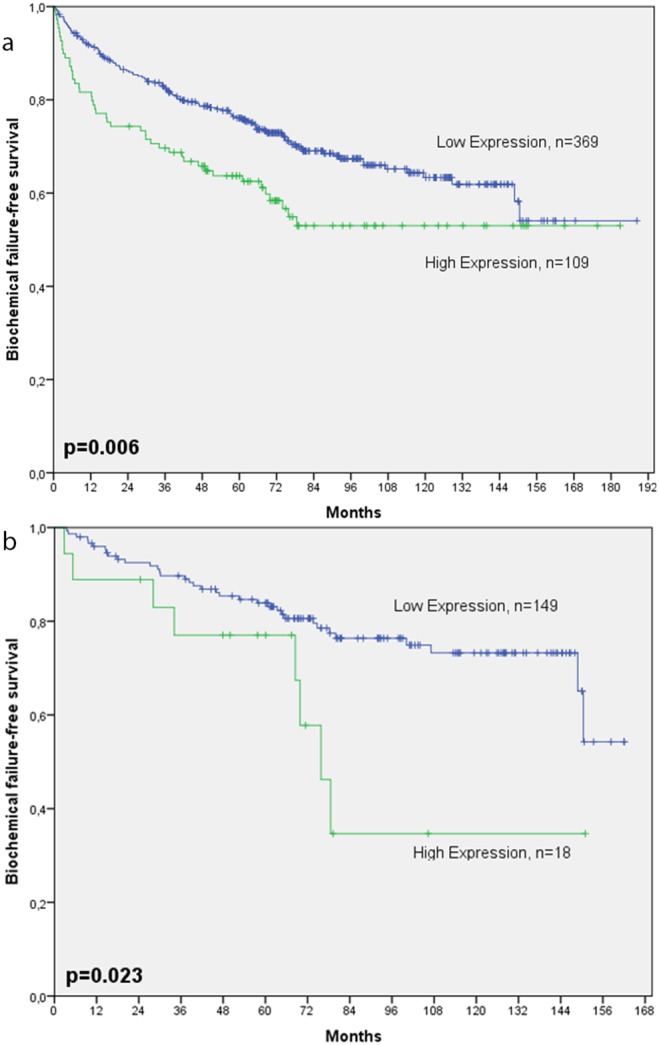
Disease-specific survival according to biochemical failure and high stromal expression of miR-21 in: a) Total cohort. **b) Patients with Gleason score 6.**

The 4^th^ quartile was used as cut-off. A high expression of miR-21 in tumor stromal areas was significantly correlated with BF (n = 170, p = 0.006, Figure 3a), and CF (n = 36, p = 0.041, figure not shown.) There was no correlation between high stromal expression of miR-21 and PCD (n = 14, p = 0.505).

In subgroup analyses we found high stromal expression of miR-21 to be significantly associated with increased risk for BF in patients with GS 6 (n = 167, p = 0.023, Figure S3b), but this were not found for patients with GS 7 (n = 262, p = 0.228), Gleason grade 3+4 (n = 189, p = 0.0290, Gleason grade 4+3 (n = 73, p = 0.818), and GS >7 (n = 36, p = 0.895). We also found high stromal expression correlated to BF for the patients with positive circumferential margins (n = 139, p = 0.026), but not for those with free circumferential margins (n = 339, p = 0.107).

### Multivariate analysis

Significant clinicopathological and molecular markers from univariate analysis were entered into the multivariate model. Table 4 presents the independent prognostic factors for BF; pT-stage (p = 0.001), Gleason grade (p = 0.021), non-apical PSM (n = 286, p = 0.001), apical PSM (n = 210, p = 0.003). For CF; Gleason grade (p = 0.013), PNI (n = 134, p = 0.028), non-apical PSM (p = 0.028).

**Table 4:** Results of Cox regression analysis of significant independent clinicopathological actors and high tumour stromal expression of mir-21 versus BF and CF.

		Biochemical Failure (BF)		Clinical Failure (CF)	
Variable		HR (95% CI)	*p*	HR (95% CI)	*p*
**pT-Stage**	pT2	1	<0.001	Not Significant	
	pT3a	1.7 (1.1–2.5)	0.011		
	pT3b	2.6 (1.6–4.2)	<0.001		
**Preop PSA**		1.4 (1.0–1.9)	0.069	Not Significant	
**Gleason**	3+3	1	0.021	1	0.013
	3+4	1.0 (0.7–1.5)	0.999	2.7 (0.9–8.6)	0.085
	4+3	1.5 (0.9–2.4)	0.125	3.5 (1.0–12.1)	0.047
	8–10	1.9 (1.3–2.7)	0.008	6.6 (2.1–21.2)	0.002
**Perineural infiltration**		Not Significant		2.3 (1.1–4.7)	0.031
**Vascular infiltration**		Not Significant		Not Significant	
**Apical PSM**		0.6 (0.4–0.8)	0.003	Not Significant	
**Non-apical PSM**		1.9 (1.3–2.7)	<0.001	2.9 (1.4–6.1)	0.004
***hsa*** **-miR-21**		1.4 (1.0–1.9)	0.089	Not Significant	

High stromal expression of miR-21 was an independent prognostic factor for BF in patients with Gleason score 6 (n = 167, HR 2.40, CI 95% 1.06–5.49, p = 0.037). A significant association between high stromal expression of miR-21 and BF was also found in the subgroup of patients with PSM (HR 1.95, CI 95% 1.95–3.21, p = 0.008). However, for the entire cohort (n = 471), there was only a trend towards an independent association between stromal expression of miR-21 and BF (n = 170, p = 0.089). High stromal expression of miR-21 was not an independent prognostic variable for CF (n = 36, p = 0.395).

## Discussion

In this study, we found a higher expression of miR-21 in cancer tissues compared with normal prostatic tissue. The expression was highest in tumor stromal areas. High tumor stromal expression of miR-21 was an independent prognostic factor for biochemical failure in patients with Gleason grade 6 but not clinical failure, probably due to few events in the latter group. To our knowledge, this is the first study reporting tumor stromal expression of miR-21 as a prognostic marker for BF after radical prostatectomy. Moreover, we also found a high stromal expression to be an independent marker for PSM in RP specimens.

Recent studies have shown that miRNAs are significantly altered in prostate cancer, suggesting that miRNAs act as key regulators of prostate carcinogenesis. Several studies have been conducted to identify the PC-specific miRNA signature, but n consensus has been reached with respect to miRNAs role in development and progression of PC [20], [21]. In a study by Violinia et al. [22], total RNA was extracted from 363 solid cancers, including prostate cancer, and 177 normal tissues. They found a general up-regulation of 39 miRNAs, including miR-21, whereas 6 miRNAs were down-regulated. These results were in partial agreement with a study by Ambs et al. in which total RNA extracted from 60 micro-dissected PC and 16 surrounding non-tumor tissues were analyzed [23]. MiR-21 is generally considered an oncogene, but so far its role in PC is unclear and the reports have been conflicting [11], [12], [14], [20]. miR-21 has been found to be elevated in PC3 and DU145 androgen-independent cell lines [24]. Moreover, miR-21 was identified as an androgen receptor-regulated miRNA whose level was elevated in PC compared with adjacent normal tissue [12]. Inhibitions of miR-21 diminish androgen-induced PC cell proliferation, whereas elevated expression of miR-21 promotes enhanced tumor growth and castration resistance *in* *vivo* [12]. Others have also found miR-21 up-regulated in patients with hormone- and chemoresistant PC [13], [15].

We found that a high tumor stromal expression of miR-21 in tumors with Gleason score 6 predicted BF. This is in line with previous reports [25]. Stromal miR-21 expression analysis may be a potential tool to predict which highly differentiated tumors that is most likely to progress. Recent studies have provided valuable insights in clarifying the involment of miR-21 in tumor microenvironment: Bullock et al. [26] demonstrated that upregulated miR-21 expression occurs in cancer-associated stromal cells but not in colo-rectal cancer cells. Moreover, they found that ectopic miR-21 expression in fibroblasts modulated the cytotoxic impact of Oxaliplatin (chemotherapheutic used in treatment of colon cancer) which resulted in cancer progression. Bronisz et al. [27] demonstrated that downregulation of mir-320 in mammary stromal fibroblasts reprograms the tumor microenvironment by activating a pro-oncogenic secretome, and interestingly, Yao et al. [28] reported that myofibroblast transdifferentiation from progenitor fibroblasts in response to TGF-β could be prevented using specific antisense inhibitors of miR-21. Together, these data suggest pro-metastatic influence of mRNAs in fibroblast differentiation and phenotype, and that miR-21 may be mediated through the tumor microenvironment. However, biological and functional evidence to support these findings, especially prostate carcinomas, is limited.

It is well known that less than 3% of patients with Gleason score≤6 will ever progress whether treated or not, and that a substantial percentage of these patients continue to undergo unnecessary treatment following a diagnosis of low risk PC (based on a PSA<10 ng/mL and stage ≤ T2a) [29], [30]. Stromal miR-21 expression analysis may be a potential tool to predict which highly differentiated tumors that is most likely to progress. Further studies to clarify the exact role of miR-21 in these highly differentiated tumors are needed. In contrast to previous reports, we found high expression of miR-21 in patients with positive surgical margins, [25]. The molecular mechanisms for this are unknown.

The divergent results of miR-21 in different studies might reflect the heterogeneity of PC, as well as different study design and methodology. A miRNA screening of the entire cohort would have strengthened our study. Besides, quality of the tissues examined (including handling) can drastically affect the interpretation of microarray data. Besides the interesting data on BF, our study is somewhat limited by the low number of cases with clinical relapse or PCD. Further studies of this microRNA and its associated pathways may uncover new mechanisms for cancer progression and therapeutic intervention.

## Conclusion

This study on miRNA profiling and validation in prostate cancer adds further evidence for miR-21 as a prognostic marker for PC. These results indicate that stromal expression of miR-21 has an important role in disease progression, but the underlying mechanism needs further investigation.

## Supporting Information

File S1 (ZIP)
